# Advanced Sensing, Fault Diagnostics, and Structural Health Management

**DOI:** 10.3390/s22239087

**Published:** 2022-11-23

**Authors:** Yongbo Li, Bing Li, Jinchen Ji, Hamed Kalhori

**Affiliations:** 1 School of Aeronautics, Northwestern Polytechnical University, Xi’an 710072, China; 2 School of Mechanical and Mechatronic Engineering, Faculty of Engineering and IT, University of Technology, Sydney, P.O. Box 123, Broadway, NSW 2007, Australia; 3 School of Mechanical and Mechatronic Engineering, The University of Technology Sydney, Sydney, NSW 2007, Australia

Advanced sensing, fault diagnosis, and structural health management are important parts of the maintenance strategy of modern industries. With the advancement of science and technology, modern structural and mechanical systems are becoming more and more complex. Due to the continuous nature of operation and utilization, modern systems are heavily susceptible to fault. Hence, the operational reliability and safety of the systems can be greatly enhanced by using the multifaced strategy of designing novel sensing technologies and advanced intelligent algorithms, and constructing modern data acquisition systems and structural health monitoring techniques. As a result, this research domain is receiving a significant amount of attention from researchers in recent years. Furthermore, the research findings have been successfully applied in a wide range of fields such as aerospace, manufacturing, transportation and processes. 

This Special Issue of *Sensors* aims to collect the latest research results and developments encompassing all the areas of advanced sensor design, fault diagnosis, and structural health management. This collection contains 10 papers that represent state-of-the-art technology developed for reliability engineering. 

Addressing the challenges of maintenance at high altitudes, Guo et al. [1] designed a novel intelligent technique for detecting the rust on transmission fitting lines with the help of unmanned aerial vehicles (UAVs). A novel convolutional neural network (CNN), namely, the R-CNN algorithm, was proposed to extract rich information about the transmission line from the UAV images. Secondly, a feature enhancement technique was added after the pooling layer of the region of interest (ROI) to enhance the feature representations of the regions that have real fittings. 

Aiming to improve the interpretability aeromagnetic data by compensating for the on-board electronic interference (OBE), a data-driven OBE interference compensation method was proposed by Wang et al. [2]. Unlike existing linear algorithms, the proposed method can reduce OBE interference without relying on any reference sensors. A long short-term memory (LSTM) network was combined with wavelet decomposition for detecting and predicting OBE interference and, subsequently, local variations of the magnetic field were estimated to remove the drift of the interference.

A novel crack monitoring technique for engineered cementitious composite (ECC) beams reinforced with hybrid bars using piezoceramic-based smart aggregates was designed by Qian et al. [3]. The designed ECC bars were fabricated and tested under the influence of cyclic loading. Two smart aggregate (SA)-based active sensing methods pasted onto both ends of the beams were used as the actuator and sensor to monitor the growth of the damage. Furthermore, a novel self-repairing index for monitoring the self-repairing capacity was proposed. 

Wang et al. [4] proposed an evaluation method using hierarchical analysis based on a combination of expert industry opinions for XLPE cables utilized in coal mines. After classifying the mining cable health status according to the degree of severity, a cloud model theory was utilized to transform the standard status level into a visualized status space. The membership degrees of each quantitative and qualitative index was calculated. An improved analytic hierarchy process (AHP) was utilized to calculate the weight of each indicator in the indicator layer. The cable health status was judged after fusing the indicator membership and the weight via the D-S evidence theory. 

A novel technique for impact calibration of a wide-range triaxial force transducer was proposed by Wang et al. [5] using the Hopkinson bar technique. In the proposed technique, the reference input force for the transducer was generated by the Hopkinson bar. Furthermore, different from the existing methods, the transverse sensitiveness of the triaxial transducer was given importance in the proposed method. The calibration results expressed by sensitivity coefficients were linearly fit by using the least square method (LSM) in a sensitivity matrix. 

Targeting the limitations of the conventional velocity stress-dependent acoustoelastic effect for short bolts, a stress-dependent attenuation estimation model was developed by Fu et al. [6]. The effect of axial stress on ultrasonic scattering attenuation was investigated by calculating the change in the energy attenuation coefficient of ultrasonic echoes after the application of an axial preload. Additionally, to overcome the challenge of obtaining a frequency-dependent attenuation coefficient, the bandwidth of the measured echoes was divided into several frequency bands to select the frequency band sensitive to the axial stress changes. Under 20-step axial preloads, the final estimation model between the axial stress and energy attenuation coefficient in the frequency band was established. 

Xie et al. [7] studied the combined effect of corrosion and crack defects on the growth of cracks in pipelines. The interaction effect of corrosion on the fatigue crack was obtained by studying the stress intensity factor (SIF) interaction impact ratio after calculating the SIF by using finite element models. One direct and one indirect approach based on extreme gradient boosting (XGboost) had been used to predict the SIF interaction impact ratio. Finally, a crack propagation model was designed based on the XGboost models, and the Paris law and corrosion growth model was proposed for pipelines with interacting crack and corrosion defects. 

Aiming to solve the limitations of prognosis operation for Francis turbine units (FTUs) in practical engineering environments, an ensemble prognostic method under variable operating conditions was proposed by Duan et al. [8]. After constructing the running data set with the help of values of the water head, active power, and vibration amplitude of the top cover, a density-based spatial clustering of applications with noise (DBSCAN) was introduced for filtering the raw data from outliers and singularities. From the cleaned raw data, a healthy state model was constructed from a Gaussian mixture model, and subsequently, a performance degradation indicator was calculated by using the negative log-likelihood. Finally, based on the designed indicator, a multiobjective prediction model was proposed based on the non-dominated sorting genetic algorithm and Gaussian process regression. 

Zhang et al. [9] analysed the nonlinear dynamic behaviour of tethered satellite formation systems in tethered space systems (TSSs) based on a simplified rigid-rod tether model. Two new stability control laws were proposed based on the tether release rate and tether tension for controlling the variation of tether length. In addition, based on the Floquet theory proposed in 1868, the periodic stability of the post-deployment time-varying control system was analysed. 

Focusing on the improvement of the sampling performance of a wireless sensor network (WSN) in real-time situations, Ha et al. [10] proposed an optimal remote monitoring system platform for SHM, which is based on the pulsed eddy current (PEC). The proposed method was utilized for measuring the corrosion of a steel-framed structure. A new circuit was designed to delay the PEC response to tackle the fast-varying output signal in an actual scenario for an efficient sampling performance. 

All the Editors would like to extend their gratitude to the authors for their significant and noteworthy contributions. We also give our sincere thanks to all the anonymous reviewers who took their time and provided feedback to the authors for the improvement of their research. Additionally, we want to thank the publishers and all the members of the *Sensors* board for providing this opportunity to present these works. 
